# A case report of cholinergic rebound syndrome following abrupt low-dose clozapine discontinuation in a patient with type I bipolar affective disorder

**DOI:** 10.1186/s12888-019-2055-1

**Published:** 2019-02-19

**Authors:** Andrea Galova, Patricia Berney, Jules Desmeules, Ioannis Sergentanis, Marie Besson

**Affiliations:** 1 Psychopharmacology Unit, Clinical pharmacology and toxicology division, Acute Medicine Department, Geneva University Hospital, Geneva, Switzerland; 2 Clinical pharmacology and toxicology division, Acute Medicice Department, Geneva University Hospital, Geneva, Switzerland; 3 Liaison Psychiatry and Crisis Intervention Unit, Psychiatry department, Geneva University Hospital, Geneva, Switzerland

**Keywords:** Withdrawal syndromes-cholinergic rebound syndrome-low dose clozapine -bipolar affective disorder-Pharmacodynamic properties-overlapping switch strategies-case report

## Abstract

**Background:**

Rebound cholinergic syndrome is a rare, but well known unwanted phenomenon occurring after abrupt clozapine discontinuation. There have been previous reported cases of cholinergic rebound in the literature; however, these reports described cholinergic rebound following cessation of high doses of clozapine in patients diagnosed with schizophrenia. Here, we report a case of rebound cholinergic syndrome and catatonia in a male patient three days after abrupt discontinuation of 50 mg of clozapine prescribed for type I bipolar affective disorder.

**Case presentation:**

A 66-year old male of Spanish origin, treated for type I bipolar affective disorder for 15 years and for Crohn disease, was brought to the emergency department because of a sudden onset of mutism, dysphagia and trismus. He was described catatonic and presented hypertension, tachycardia and tachypnea. His body temperature was normal and the laboratory tests were unremarkable at presentation. A head CT and an EEG were in the normal range.

While reviewing his history, it appeared the he was on clozapine 50 mg a day, first introduced 2 months ago, during a previous hospitalization for a manic episode resistant to other mood stabilizers. For an unknown reason, the patient’s psychiatrist stopped clozapine three days before the admission and replaced it by risperidone 5 mg and quetiapine 200 mg daily. A cholinergic rebound syndrome was then evoked. The patient’s ability to speak recovered dramatically and fast after the intravenous administration of 2.5 mg of biperiden supporting the diagnosis. Risperidone and quetiapine were also stopped. The patient fully recovered in 20 days after the reintroduction of 50 mg of clozapine and 2.5 mg of biperiden daily.

**Conclusions:**

This case report underscores that cholinergic rebound syndrome may occur in patients suffering from bipolar affective disorders, being on clozapine as a mood stabilizer. The low dose clozapine does not preclude severe manifestations of the phenomenon. Progressive tapering should therefore be adopted in any case.

## Background

Withdrawal syndromes are emergent drug adverse events, due to receptor supersensitivity elicited by a sudden drug discontinuation [1]. Such adverse events are well described and easy recognizable when tapering down and stopping a wide range of psychotropic medication. However, withdrawal syndromes may also occur when switching from one molecule to another and may be then more difficult to diagnose.

Dopamine and serotonin supersensitivity, as well as cholinergic rebound syndrome, all belong to the well described antipsychotic withdrawal syndromes. Special attention should be paid when the switching molecules do not share the same pharmacodynamic or pharmacokinetic properties. Overlapping “plateau” switch strategies, or transient prescription of benzodiazepines or anticholinergic drugs when an abrupt discontinuation is unavoidable, should be adopted [2].

Cholinergic rebound syndrome is induced in susceptible patients after an abrupt discontinuation of a drug that blocks muscarinic acetylcholine receptors. Its central component is characterized by agitation, confusion, psychosis, anxiety, insomnia, sialorrhea and extrapyramidal manifestations. Diarrhea, sweating, nausea (with or without vomiting) and signs of dysautonomia are part of the peripheral component [2].

Clozapine is a second generation antipsychotic, which blocks multiple receptors, including the dopamine D2 receptors, the serotonin 5-HT 2A and 5-HT2C receptors, histamine H1, adrenergic α1 and M1 to M5 muscarinic receptors [1, 3]. Given its unique high affinity for muscarinic receptors, clozapine is at a high risk of cholinergic rebound syndrome on cessation or when switching for another antipsychotic [3–5].

Several case reports of cholinergic rebound syndrome have been published, after abrupt withdrawals in schizophrenic patients needing high doses of clozapine (from 300 to 900 mg daily) [4, 6–11].

Besides, an alert to the clinicians was published shortly after the commercialization of risperidone, warning that cholinergic rebound syndrome may occur when clozapine was switched to risperidone, given the difference of binding affinity on muscarinic receptors between the two compounds [12]. Moreover two cases of a severe cholinergic rebound syndrome after a clozapine-olanzapine switch in schizophrenic patients were reported [13]. This may be somehow surprising since olanzapine is also considered to have a significant affinity for muscarinic receptors. However works comparing in vitro affinity on muscarinic receptors of atypical antipsychotics showed that the anticholinergic activity of olanzapine was less pronounced than the clozapine anticholinergic activity by a factor ten [14]. This difference as well as administered doses may have accounted for the observed withdrawal syndromes.

Herein, we report a case of cholinergic rebound syndrome due to abrupt cessation of a low dose of clozapine (50 mg daily), prescribed for type I bipolar affective disorder. We would like to highlight the importance of early clinical recognition of the syndrome as it may be hindered by heterogeneous clinical presentations, frequently overlapping with other drug syndromes or adverse effects. Early recognition is obviously essential, since the treatment is specific and usually rapidly successful.

## Case presentation

A 66-year old male of Spanish origin, known for Crohn disease and type I bipolar affective disorder for at least 15 years, was brought to the emergency department for sudden mutism that developed within 2 h. In the emergency room, the patient was described catatonic, presenting with no speech at all, staring, stupor, immobility and rigidity of the four extremities and a trismus. He was not vomiting. Standardized scale for catatonia was not used on admission. Retroactively, according to the psychiatrist in charge of the initial evaluation of the patient, the score on Bush-Francis catatonia scale would have been 8.

The patient was diaphoretic, with obvious dysautonomic signs: blood pressure was 175/126 mmHg, heart rate was 105 bpm and respiratory frequency was 22/min. His temperature was 36.7 °C. Laboratory results showed a mild hyponatremia (Na^+^ 132 mmol/l, N: 136–144), the glomerular filtration rate was 95 ml/min and liver tests were as follow: ASAT 33 U/l (N: 14–50), ALAT 30 U/l (N: 12–50), alkaline phosphatase 61 U/l (N: 25–102), γGT 62 U/l (N: 9–40). Creatine kinase (CK) blood concentration was 56 U/l (N: 47–222) and arterial gasometry was in the normal ranges. Hematology was unremarkable, namely leucocytes were 4.9 G/L, with 79% neutrophils. A brain CT excluded an ischemic stroke and a cerebral hemorrhage. A 24 h-EEG was unremarkable. Clozapine blood level at admission was not determined. While reviewing the patient history, it appeared that he was hospitalized in a psychiatric ward until 2 months ago for a manic episode, which has required many drug treatment trials because of adverse effects or treatment failure. These were namely, a three-fold CK elevation related to olanzapine, mild leucopenia and agranulocytosis related to aripiprazole, extrapyramidal manifestations on haloperidol and high dose quetiapine lack of efficacy. Facing this resistant episode a try with clozapine was then made. The patient was eventually discharged, stabilized on clozapine 50 mg daily, with 12.5 mg on demand, valproic acid 500 mg twice daily and his usual treatment for Crohn disease (mesalazine 1000 mg, azathioprine 50 mg twice daily).

For an unknown reason, clozapine was then abruptly stopped and replaced by risperidone 5 mg daily associated with quetiapine 200 mg daily three days before the emergency admission. Besides valproid acid was decreased to 300 mg daily.

Therefore, the hypothesis of a cholinergic rebound syndrome was evoked, with a potential contribution of risperidone overdose in the catatonic manifestation. Risperidone and quetiapine were stopped and biperidene 2.5 mg intravenously was administrated leading to a dramatic recovering of the patient’s ability to speak within minutes after application. Other manifestations (nausea, sweating, tachycardia, hypertension) took few days to recover on clozapine 50 mg and daily oral administration of 2.5 mg of biperiden. The patient was eventually discharged twenty days after admission with only mild bradykinesia and gait instability. His mental state was stable during the whole hospital stay.

## Discussion and conclusion

The clinical presentation on admission oriented the differential diagnosis toward a neurological condition such as a stroke, an intracerebral hemorrhage or epilepsy. They were excluded by a brain CT and a 24 h-EEG. The remaining possibility was a drug related condition, namely neuroleptic malignant syndrome, risperidone overdose or cholinergic rebound syndrome. Neuroleptic malignant syndrome was eventually not retained in the absence of body temperature and CK elevation. The rapid recovery after administration of the anticholinergic biperiden and clozapine reintroduction one day after admission pointed out to a cholinergic rebound syndrome.

To the best of our knowledge, this is the first report describing a cholinergic rebound syndrome following an abrupt interruption of a low dose clozapine (50 mg only) prescribed for bipolar affective disorder. Due to clozapine high affinity for muscarinic receptors, cholinergic rebound syndrome is a well-known emergent adverse event but traditionally considered in schizophrenic patients on high dose [4, 6–9, 11]. According to a previous study evaluating clozapine 200 mg daily for at least a month abrupt withdrawal, half of the 28 schizophrenic patients developed mild withdrawal symptoms. They included agitation, headache and nausea [8]. In addition, 20% (five patients) presented with moderate to severe withdrawal symptoms (nausea with vomiting, diarrhea, psychosis) needing specialized care. Manifestations took place within 24 h to 3 days after clozapine withdrawal.

Our case report underscores that cholinergic rebound syndrome may occur the same way in patients suffering from bipolar affective disorders, having clozapine as a mood stabilizer. Moreover, the low dose of clozapine does not preclude severe manifestations of cholinergic rebound syndrome. Therefore, progressive tapering must be adopted in any case.

Clozapine plasma concentration was not determined on admission, hence a drug-drug interaction, accounting for higher values than expected according to the dose, could not be definitely excluded. However, the only cytochrome p450 (CYP) blockers found in the patient treatment on admission was valproic acid that is a strong CYP 2C9 inhibitor. Clozapine main metabolite pathway is CYP 1A2 whereas CYP 2C9 is only a minor route [15]. A clinically significant interaction was therefore not strongly expected.

Signs of catatonia at initial clinical presentation, was confounding as it does not belong to the classical description of cholinergic rebound syndrome [2]. However, several cases of a cholinergic rebound syndrome or clozapine withdrawal syndrome manifested with catatonia as the main feature have been described in the literature [11, 16–22]. Catatonia is a complex phenomenon that has been associated with a wide range of medical conditions. Drug-related catatonia is classically linked to dopamine receptor blockade, whereas drug-withdrawal catatonia are frequently described after benzodiazepines and clozapine withdrawal [23]. Given the complex interaction between the dopamine and the cholinergic systems in motor function regulation [24] and the use of anticholinergic medication to counterbalance the effect of dopamine blockade, a sudden cholinergic overdrive may be advocated as an explanation to clozapine withdrawal catatonia**.**

Another point worth mentioning is a possible participation of a risperidone overdose to the extrapyramidal features as well as the autonomic instability presented by the patient (dysphagia, trismus and rigidity of the four extremities). The introduction of 5 mg of risperidone was abrupt instead of slowly titrated in this patient, who has developed extrapyramidal manifestations on haloperidol in the past. Moreover, according to different sources and clinical experience, 0,5 to 2 mg of the risperidone would have been a more appropriate equivalent dose to 50 mg clozapine, based on their antipsychotic activity [25]. Since clinical manifestations of exaggerated dopamine blockade and cholinergic rebound are overlapping and since the efficacy of anticholinergic drug administration to counteract the effect of dopamine blockade is in some cases well described [26, 27], the exact contribution of both phenomenon is undeterminable and remains subject to interpretation.

In conclusion, we presented the first case of severe cholinergic rebound syndrome due to a low dose clozapine abrupt withdrawal administered as a mood stabilizer. This case highlights the need of being aware of the pharmacodynamic properties of psychotropic drugs, especially since their indications broaden.

The psychotropic drug associated syndromes or adverse events are overlapping. Clinicians should consider drugs pharmacodynamic properties when switching or stopping psychotropic medications. It would allow a rapid recognition of sometimes puzzling clinical manifestations and rapid introduction of the appropriate treatment.

Clozapine cholinergic rebound syndrome may be prevented by systematically adopting the overlapping “plateau” switch strategies, when switching from clozapine to another antipsychotic due to its unique muscarinic affinity. When abrupt discontinuation is needed, in case of severe agranulocytosis for example, the introduction of an anticholinergic agent is recommended. Trihexyphenidyl (1 mg per 40 mg clozapine), benztropine (4 mg) [28] and atropine (1 mg) [21] have been described. Biperiden was chosen in our case because it is the only oral anticholinergic registered in Switzerland. If clozapine-withdrawal symptoms, namely catatonia, mania or rebound psychosis are resistant to progressive tapering or anticholinergic substitution, electroconvulsive therapy may be proposed according to some authors [29].

## Abbreviations

bpm: beats per minute
CK: creatine kinase
CT: computed tomography
CYP: cytochrome P450
EEG: electroencephalography
GABA: gamma-amino butyric acid
mm Hg: millimetre of mercury
